# Combined dietary folate, vitamin B-12, and vitamin B-6 intake influences plasma docosahexaenoic acid concentration in rats

**DOI:** 10.1186/1743-7075-9-49

**Published:** 2012-05-30

**Authors:** Nick van Wijk, Carol J Watkins, Robert J J Hageman, John C W Sijben, Patrick G H J Kamphuis, Richard J Wurtman, Laus M Broersen

**Affiliations:** 1 Nutricia Advanced Medical Nutrition, Danone Research, Centre for Specialised Nutrition, PO Box 7005, 6700 CA, Wageningen, The Netherlands; 2 Department of Brain and Cognitive Sciences, Massachusetts Institute of Technology, Cambridge, MA, USA; 3 Utrecht Institute for Pharmaceutical Sciences (UIPS), Utrecht University, Utrecht, The Netherlands

**Keywords:** B-vitamins, Plasma DHA, Plasma homocysteine, Methylation capacity, Rats

## Abstract

**Background:**

Folate, vitamin B-12, and vitamin B-6 are essential nutritional components in one-carbon metabolism and are required for methylation capacity. The availability of these vitamins may therefore modify methylation of phosphatidylethanolamine (PE) to phosphatidylcholine (PC) by PE-*N*-methyltransferase (PEMT) in the liver. It has been suggested that PC synthesis by PEMT plays an important role in the transport of polyunsaturated fatty acids (PUFAs) like docosahexaenoic acid (DHA) from the liver to plasma and possibly other tissues. We hypothesized that if B-vitamin supplementation enhances PEMT activity, then supplementation could also increase the concentration of plasma levels of PUFAs such as DHA. To test this hypothesis, we determined the effect of varying the combined dietary intake of these three B-vitamins on plasma DHA concentration in rats.

**Methods:**

In a first experiment, plasma DHA and plasma homocysteine concentrations were measured in rats that had consumed a B-vitamin-poor diet for 4 weeks after which they were either continued on the B-vitamin-poor diet or switched to a B-vitamin-enriched diet for another 4 weeks. In a second experiment, plasma DHA and plasma homocysteine concentrations were measured in rats after feeding them one of four diets with varying levels of B-vitamins for 4 weeks. The diets provided 0% (poor), 100% (normal), 400% (enriched), and 1600% (high) of the laboratory rodent requirements for each of the three B-vitamins.

**Results:**

Plasma DHA concentration was higher in rats fed the B-vitamin-enriched diet than in rats that were continued on the B-vitamin-poor diet (*P* = 0.005; experiment A). Varying dietary B-vitamin intake from deficient to supra-physiologic resulted in a non-linear dose-dependent trend for increasing plasma DHA (*P* = 0.027; experiment B). Plasma DHA was lowest in rats consuming the B-vitamin-poor diet (*P* > 0.05 vs*.* normal, *P* < 0.05 vs*.* enriched and high) and highest in rats consuming the B-vitamin-high diet (*P* < 0.05 vs*.* poor and normal, *P* > 0.05 vs*.* enriched). B-vitamin deficiency significantly increased plasma total homocysteine but increasing intake above normal did not significantly reduce it. Nevertheless, in both experiments plasma DHA was inversely correlated with plasma total homocysteine.

**Conclusion:**

These data demonstrate that dietary folate, vitamin B-12, and vitamin B-6 intake can influence plasma concentration of DHA.

## Background

Several clinical studies in different populations have found a negative correlation between serum, plasma or erythrocyte content of docosahexaenoic acid (DHA) and markers of B-vitamin deficiency such as plasma levels of homocysteine and/or *S*-adenosylhomocysteine (SAH) [1-3]. In line with these observations, dietary deficiency studies in rats have shown that deficiencies of folate, vitamin B-12, or vitamin B-6 may reduce peripheral DHA levels [4-6]. Although these data indicate a link between dietary B-vitamin intake and DHA status, it has not been studied whether concurrently varying the dietary intake of folic acid, vitamin B-12, and vitamin B-6 could influence plasma DHA concentration.

Hypothetically, dietary B-vitamin availability might influence plasma DHA by influencing synthesis of phosphatidylcholine (PC) in the liver. Hepatic PC can be synthesized by two different metabolic pathways, the cytidine diphosphate (CDP)-choline pathway (Kennedy cycle) and the phosphatidylethanolamine-*N*-methyltransferase (PEMT) pathway. The CDP-choline pathway utilizes 1,2-diacylglycerol and CDP-choline for the synthesis of PC [7], whereas PEMT catalyzes the sequential methylation of phosphatidylethanolamine (PE) to PC [8]. It has been suggested that the methylation of PE to PC by PEMT plays an important role in the transport of polyunsaturated fatty acids (PUFAs) like DHA from the liver to the plasma and other tissues [3,9,10]. Most likely, two mechanisms are involved. First, PC synthesis is required for normal secretion of very low density lipoprotein (VLDL) from liver cells [11]. An impairment of the PEMT pathway results in a diminished PC synthesis and therefore limits hepatic secretion of VLDL [12]. Because VLDL is the main carrier of endogenous triglycerides, phospholipids, and cholesterol esters, impairment of the PEMT pathway directly affects the transport of these components from the liver to peripheral tissues [13]. Second, PC synthesized by the PEMT pathway contains more PUFAs such as DHA than does PC synthesized by the CDP-choline pathway because PEMT prefers species of PE containing PUFAs [10,14,15]. Hence, PEMT activity can influence both VLDL secretion and the rate of synthesis of PUFA-rich PC species. Therefore, a factor that influences hepatic PEMT activity could potentially affect the availability of PUFAs such as DHA in plasma [9] and even their transport to the brain [16].

Folate, vitamin B-12, and vitamin B-6 availability are important determinants of methionine and *S*-adenosylmethionine (SAM) synthesis and of SAH and homocysteine clearance, and therefore of methylation capacity. Thus, B-vitamin availability can be hypothesized to directly modify liver PEMT activity and PEMT-dependent PUFA secretion. This in turn, is predicted to influence plasma PUFA concentration and thus tissue availability. If so, then adequate dietary intake of these three vitamins would be necessary to maintain normal plasma DHA concentrations, and increasing dietary intake above the normal range might be expected to increase plasma DHA. The aim of the present study was to determine whether dietary enrichment with the combination of these three B-vitamins could increase plasma DHA concentration in rats. In a first experiment, plasma DHA concentration was measured in rats that had consumed a B-vitamin-poor diet for 4 weeks, after which they were either continued on the B-vitamin-poor diet or switched to a B-vitamin-enriched diet for another 4 weeks. In a second experiment, the dependency of plasma DHA on dietary B-vitamin content was determined by feeding rats for 4 weeks one of four diets containing varying levels of the B-vitamins across the range from inadequate to supra-physiological.

## Methods

Two experiments were conducted to investigate the effects of varying dietary levels of folate, vitamin B-12, and vitamin B-6 on plasma DHA concentration. Experiment A was conducted at the Department of Brain and Cognitive Sciences, Massachusetts Institute of Technology (Cambridge, MA, USA). Experiment B was conducted at the Centrum Kleine Proefdieren, Wageningen University (Wageningen, The Netherlands).

### Animals

A total of sixty-four male Sprague–Dawley rats (Crl:CD(SD)) were obtained from either Charles River, Wilmington, MA, USA (experiment A; *n* = 16) or Charles River, Sulzfeld, Germany (experiment B; *n* = 48). Animals aged 6–8 weeks on arrival were housed in groups in a temperature- and light-controlled room, under 12 h light–12 h dark cycles. Rats had free access to food and water. Body weight was registered once a week. All animal experimental protocols were conducted in accordance with international and national laws and institutional guidelines and approved by the local ethics committee, i.e. the Committee on Animal Care at Massachusetts Institute of Technology, Cambridge, MA, USA (experiment A) and DEC Consult, Bilthoven, The Netherlands (experiment B).

### Diets

Four different diets with increasing folate, vitamin B-12, and vitamin B-6 contents were used: 1) B-vitamin-poor; 2) B-vitamin-normal; 3) B-vitamin-enriched; and 4) B-vitamin-high. Diets were AIN-93 M based [17], isoenergetic, and identical with respect to their protein, carbohydrate, fat, fiber, and mineral contents. All diets were devoid of any measurable amounts of DHA. The vitamin mix (AIN-93-VX) [17] was prepared without folic acid, cyanocobalamin, and pyridoxine; these vitamins were subsequently supplemented accordingly. Diets were formulated with vitamin-free, ethanol-precipitated casein (Harlan Teklad, Madison, WI, USA) and were manufactured by Research Diet Services, Wijk bij Duurstede, The Netherlands (experiment A) and Ssniff Spezialdiäten, Soest, Germany (experiment B).

The B-vitamin-poor diet contained low amounts of folate (<0.1 mg/kg), vitamin B-12 (<1.0 μg/kg), and vitamin B-6 (<0.6 mg/kg). No sulfathiazole drugs were added to the diet and therefore a limited amount of folate was still expected to be provided by the gut flora. Vitamin B-12 deficiency in the rat is difficult to achieve because of considerable endogenous storage of this vitamin. To attain a moderate reduction of endogenous vitamin B-12, the B-vitamin-poor was supplemented with 50 g/kg pectin (polygalacturonic acid, high methoxyl, Obipektin®, NF/USP Citrus; TEFCO FoodIngredients, Bodegraven, The Netherlands), which binds vitamin B-12 in the intestine, making it less bioavailable [18]. Pectin consequently promotes depletion of endogenous vitamin B-12 through the enterohepatic circulation of the vitamin. Since pectin could affect food intake [19], all four diets were supplemented with pectin to maintain uniform intakes of the diets. Pectin has minimal effects on vitamin B-12 status when the diet contains adequate amounts of this vitamin [18].

The B-vitamin-normal diet, the B-vitamin-enriched diet, and the B-vitamin-high diet provided 100%, 400%, and 1600%, respectively, of the requirements for each of the three vitamins according to the National Research Council report on the nutrient requirements of laboratory animals [20]. The exact dietary levels of the three B-vitamins in each experimental diet are indicated in Table 1.

**Table 1:** Folate, vitamin B-12, and vitamin B-6 content of the experimental diets.

**Diet description**		**Calculated dietary levels**		
		**Folate (folic acid)**	**Vitamin B-12 (cyanocobalamin)**	**Vitamin B-6 (pyridoxine-HCL)**
	**% *of recommended levels*[**[20]**]**		***mg*/*kg diet***	
B-vitamin-poor	~0%	<0.1	<0.001	<0.6
B-vitamin-normal	100%	1.0	0.05	6.0
B-vitamin-enriched	400%	4.0	0.20	24.0
B-vitamin-high	1600%	16.0	0.80	96.0

### Experimental design

In experiment A, B-vitamin paucity was first induced in all rats by feeding them the B-vitamin-poor diet for 4 weeks. Subsequently, animals were either continued on the B-vitamin-poor diet or switched to the B-vitamin-enriched diet for another 4 weeks. In experiment B rats were directly fed one of the four experimental diets for 4 weeks.

### Tissue preparation

After the supplementation period, animals that had been feed-deprived for 3–4 hours were killed by CO_2_ gas inhalation (experiment A) or by inhalation of isoflurane vaporized in medicinal air (experiment B) and subsequent decapitation by guillotine. Trunk blood was collected through a funnel into EDTA-containing tubes. After centrifugation at 1750 × *g* for 10 min, plasma was aspirated and analyzed for plasma DHA and homocysteine.

### Plasma DHA and plasma total homocysteine analysis

Plasma total lipid DHA was detected using GC. Total lipid content was extracted from plasma by adding methanol and dichloromethane. Samples were subsequently centrifuged at 1750 × *g* for 10 min and the organic phase (dichloromethane and lipids) was collected. 200 μL of the dichloromethane layer was dried using a SpeedVac® concentrator. Next, 2.0 mL methanol and 40 μL concentrated sulfuric acid were added to the dried extract. The samples were heated at 100 °C for 60 min, and 2 mL hexane and 0.5 mL 2.5 mol/L sodium hydroxide solution were added. After vortexing and centrifuging the samples for 5 min at 1750 × *g*, the upper layer was collected and dried using a SpeedVac®. Dried samples were subsequently dissolved in 125 μL iso-octane and analyzed by GC using flame ionization detection.

Plasma total homocysteine was determined by fluorometric HPLC as previously described [21]. Briefly, thiol amino acids (free and protein-bound) were reduced with tri-*n*-butylphosphine. After protein precipitation and centrifugation to remove the proteins, thiol groups were derivatized with 7-fluoro-2-oxa-1,3-diazole-4-sulfonamide reagent. The content of the derivatized thiol amino acids was determined by fluorescence detection with excitation at 385 nm and emission at 515 nm.

### Statistical methods

All statistical analyses were performed using SPSS (version 15.0, SPSS Inc., Chicago, IL, USA). Data were expressed as means ± SEM. P-values <0.05 were considered significant. Effects of dietary B-vitamins on body weight were analyzed using repeated-measures ANOVA with dietary B-vitamins as between-subject factor and week as within-subject factor. Plasma DHA and homocysteine concentration were compared between rats fed the diets varying in B-vitamin content using ANOVA and post hoc comparisons were performed when appropriate. Standard Pearson correlation coefficients were calculated for plasma DHA and homocysteine.

## Results

### Experiment A

**Figure 1:**
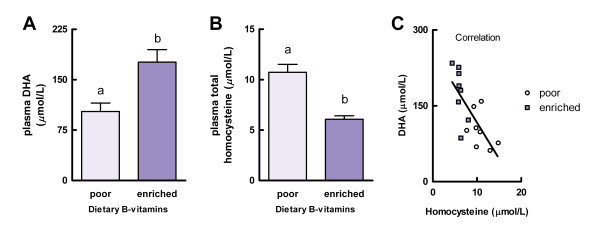
**Experiment A: effects of dietary folate, vitamin B-12, and vitamin B-6 on plasma DHA and homocysteine concentrations.** Plasma DHA (**A**) and plasma total homocysteine (**B**) concentrations and their correlation (**C**; *r* = −0.73, *P* = 0.001) in rats that received either a B-vitamin-poor diet for 8 weeks (poor) or a B-vitamin-poor diet for 4 weeks followed by a B-vitamin-enriched diet for 4 weeks (enriched). Values are means, with the SEM represented by vertical bars. Different letters indicate mean values were significantly different (*P* < 0.01, *n* = 8 per experimental group).

After feeding all animals the B-vitamin-poor diet for 4 weeks, they were randomized into the two experimental groups according to their body weight. During the 4 week intervention period body weight was unaffected by the level of dietary B-vitamins (F(1,14) = 0.77, *P* = 0.40). After the 4 week intervention period, plasma DHA concentration was 72% higher in rats fed the B-vitamin-enriched diet (176.2 ± 18.3 μM) than in rats that were continued on the B-vitamin-poor diet (102.6 ± 12.5 μM; F(1,14) = 10.97, *P* = 0.005; Figure 1A). In addition, plasma total homocysteine concentration was lower in animals receiving the B-vitamin-enriched diet (6.1 ± 0.4 μM) as compared to the B-vitamin-poor group (10.7 ± 0.8 μM) (F(1,14) = 29.35, *P* < 0.001; Figure 1B). A significant inverse correlation was observed between plasma concentrations of DHA and homocysteine (*r* = −0.73, *P* = 0.001; Figure 1C).

### Experiment B

**Figure 2:**
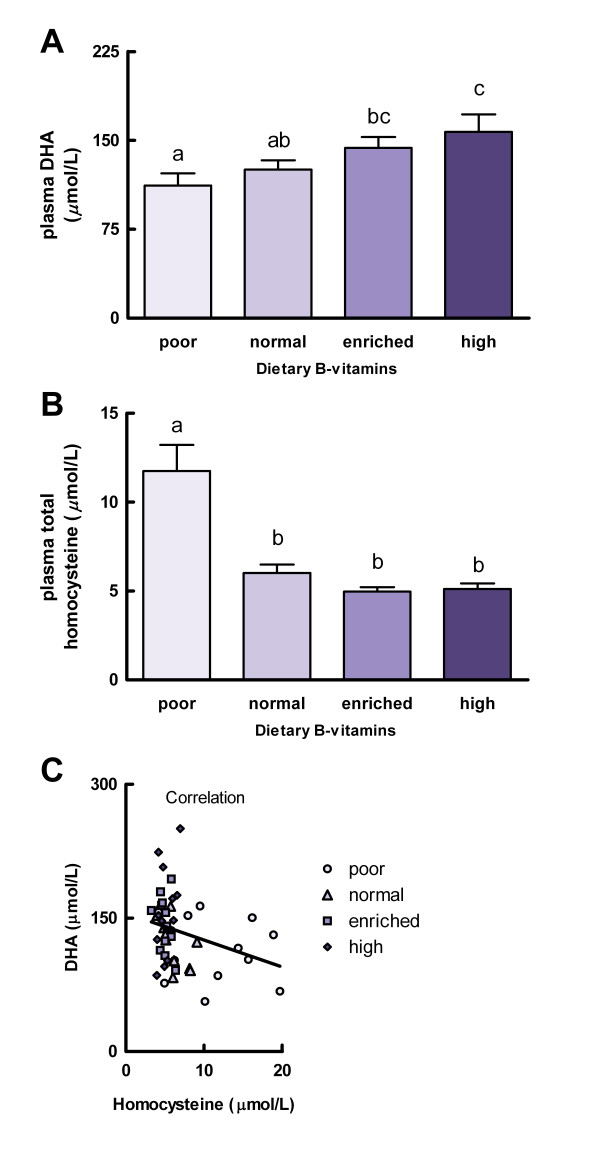
**Experiment B: dietary folate, vitamin B-12, and vitamin B-6 intake dose-dependently modifies plasma DHA concentration.** Plasma DHA (**A**) and plasma total homocysteine (**B**) concentrations and their correlation (**C**; *r* = −0.29, *P* = 0.043) in rats that received one of the four experimental diets with varying doses of folate, vitamin B-12, and vitamin B-6 for 4 weeks. Values are means, with the SEM represented by vertical bars. Different letters indicate mean values were significantly different (*P* < 0.05, *n* = 12 per experimental group).

Animals were randomized into the four experimental groups according to their body weights at the start of the intervention period. Body weight did not differ between the experimental groups fed the four different diets for 4 weeks (F(3,44) = 0.16, *P* = 0.92). After the intervention period, plasma DHA concentration was found to be dependent on dietary B-vitamin intake (F(3,44) = 3.37, *P* = 0.027; Figure 2A). Plasma DHA showed a non-linear dose-dependent relationship to dietary B-vitamin intake. Compared with rats consuming B-vitamin-normal diet (125.4 ± 8.0 μM) and B-vitamin-enriched diet (143.8 ± 9.0 μM), plasma DHA was reduced in rats consuming the B-vitamin-poor diet (111.9 ± 10.3 μM, *P* > 0.05 vs*.* normal, *P* < 0.05 vs*.* enriched) and increased in rats consuming the B-vitamin-high diet (157.1 ± 14.9 μM, *P* < 0.05 vs. normal, *P* > 0.05 vs. enriched). Dietary B-vitamin intake also affected plasma total homocysteine (F(3,44) = 16.18, *P* < 0.001; Figure 2B). Plasma homocysteine was increased in rats fed the B-vitamin-poor diet (11.7 ± 1.5 μM) as compared with rats fed the B-vitamin-normal diet (6.0 ± 0.5 μM, *P* < 0.05), whereas the B-vitamin-enriched (5.0 ± 0.2 μM) and B-vitamin-high (5.1 ± 0.3 μM) diet further reduced plasma homocysteine as compared with the B-vitamin-normal group although this reduction was not significant (*P* > 0.05 vs*.* enriched, *P* > 0.05 vs*.* high). Plasma DHA and homocysteine showed a significant inverse correlation (*r* = −0.29, *P* = 0.043; Figure 2C).

## Discussion

The present results show that concurrently varying the dietary intake of folate, vitamin B-12, and vitamin B-6, across the range from inadequate to supra-physiological supplementation, can dose-dependently modify plasma DHA concentration. Experiment A demonstrates that plasma DHA concentration is dependent on dietary intake of folate, vitamin B-12, and vitamin B-6 in rats. Rats receiving the B-vitamin-enriched diet showed higher plasma DHA and lower plasma homocysteine concentrations as compared to rats that were continued on the B-vitamin-poor diet. In experiment B the observations of experiment A were replicated and extended by showing a positive dose–response relationship between dietary B-vitamins and plasma DHA concentration. Plasma DHA and plasma homocysteine concentrations were comparable between animals fed the B-vitamin-poor diet for either 4 weeks (Experiment B) or 8 weeks (Experiment A). In addition, plasma homocysteine concentrations were only moderately increased by the B-vitamin-poor diet and therefore this diet is considered to induce only a mild B-vitamin deficiency.

It should be noted that the present experiments were conducted in different labs, under somewhat different experimental conditions (e.g. time, animal supplier, diet manufacturer, experimental design), that might have been responsible for small variations in absolute plasma DHA concentration between the two experiments. However, despite the differences in experimental conditions, the results from both experiments are highly consistent and show a robust effect of dietary B-vitamins on plasma DHA concentration.

The methylation of PE to PC by PEMT requires the methyl donor SAM. This reaction is not only influenced by the availability of SAM, but is also inhibited by SAH; the ratio of SAM to SAH therefore affects the activity of PEMT [8]. Moreover, SAH is hydrolyzed to homocysteine via a reversible reaction: thus excess homocysteine will result in increased SAH, thereby inhibiting PEMT [22]. Folate, vitamin B-12, and vitamin B-6 could enhance PEMT activity both by reducing homocysteine levels and by increasing methionine levels, resulting in an increased SAM to SAH ratio, and thus an increased methylation capacity. Both experiments revealed a significant inverse correlation between plasma homocysteine and plasma DHA concentrations. This correlation suggests that the observed effects of B-vitamins on plasma DHA concentration may be explained by the effects of these vitamins on methylation capacity and subsequent enhancement of PC synthesis by the PEMT pathway. Because PC species generated by the PEMT pathway are rich in PUFAs, especially DHA [10,14,15], and because the PEMT pathway influences secretion of VLDL from the liver [12,13], the PEMT pathway may contribute to transport of DHA from the liver to peripheral tissues. This hypothesis is supported by findings from Zeisel *et al.*, who investigated lipid metabolism in PEMT knockout mice and found diminished plasma levels of DHA in adult knockout animals [9]. In a subsequent study, brains from fetuses (embryonic day 17) derived from PEMT knockout dams had lower levels of DHA in several phospholipid species in the brain as compared to wild type controls [16]. In addition, results of a recent human study even suggest that plasma PC-DHA can be used as a marker for *in vivo* hepatic PEMT activity [23].

The present results show that concurrent dietary folate, vitamin B-12, and vitamin B-6 deficiency can lower plasma DHA concentration, which is in line with three previous experiments investigating effects of deficiencies of single B-vitamins in rats. Durand *et al.* found that dietary folate deficiency reduces relative n3 PUFA levels of the plasma total lipid fraction in rats [4]. In an early study by Peifer and Lewis, PUFA levels in PC and PE of rat liver and brain were changed by long term (20 weeks) dietary vitamin B-12 deficiency [5]. Dietary vitamin B-6 deficiency was also found to decrease the proportion of PUFAs in the phospholipid fractions of various rat tissues [6]. In addition to the observations on the effects of B-vitamin deficiency, the present results indicate that plasma DHA concentrations can be enhanced by combined supplementation with high levels of these vitamins. Pita and Delgado previously also found a rise in the proportion of DHA in various lipid fractions in plasma and other tissues after daily folate administration in rats for 15 days [24]. In that study, folate was administered intramuscularly (500 μg/kg body weight as 5-methyltetrahydrofolate). Notwithstanding the different route of administration, this intramuscular dose falls in the dietary supplementation range used in the present study, i.e. approximately 200 and 800 μg folate per kg body weight per day for, respectively, the B-vitamin-enriched and B-vitamin-high diet.

The interdependence between B-vitamin intake or status, homocysteine, and DHA status is also suggested by several studies in humans. In a multicenter trial in pregnant women, oral folate supplementation (400 μg/day) was associated with a minimal but significant increase in maternal plasma DHA level in the phospholipid faction when compared to the control subjects over time [25]. Crowe *et al.*, however, observed no change in relative DHA level in plasma PC after supplementation of folate (1000 μg/day), vitamin B-12 (500 μg/day), and B-6 (10 mg/day) for two years to older individuals [26]. The discrepancy between these results and those of the present preclinical study might be explained by the relative considerable higher dose of folate used in the present study. Additionally, analysis of the percentage of DHA in one phospholipid fraction, as was performed by Crowe *et al.,* is an essentially different parameter than the absolute DHA concentration of the total lipid fraction measured in the present experiment. In accordance with the present results, several clinical studies have shown a negative correlation between plasma, serum, or erythrocyte phospholipid DHA content and plasma levels of homocysteine [1-3], SAH [3], or SAM:SAH ratio [23]. Moreover, two clinical studies in men indicated a positive correlation either between plasma DHA concentration and erythrocyte folate level [27] or plasma phospholipid DHA concentration and serum vitamin B-12 level [1]. In the latter study, plasma phospholipid DHA did not correlate with serum folate [1].

All three B-vitamins are necessary for methylation capacity. Folate (as 5-methyltetrahydrofolate) and vitamin B-12 are cofactors in the remethylation of homocysteine to methionine, from which SAM is subsequently regenerated. Vitamin B-6 is involved in facilitating the reversible conversion of serine to glycine, which ultimately can generate 5-methyltetrahydrofolate, i.e. the methyl donor for the remethylation of homocysteine to methionine. Vitamin B-6 is also the cofactor for the transsulfuration reaction responsible for the irreversible conversion of homocysteine to cysteine, and hence the clearance of homocysteine. It can be speculated that the effects of the three B-vitamins acting together have a greater impact on PC metabolism via PEMT than each B-vitamin individually. Nevertheless, it should also be noted that the exact mechanism(s) by which the three B-vitamins could have increased plasma DHA concentration is not fully understood and that the relative contribution of PEMT activity to total plasma DHA concentration is unknown. To elucidate this, additional experiments are required in which additional parameters could be measured, e.g. plasma and tissue levels of B-vitamins, SAM, SAH, and phospholipid species.

Recently, we demonstrated that rat plasma choline concentration is also dependent on dietary intake of the three B-vitamins, possibly mediated by enhancing methylation capacity and hence PC synthesis [28]. Because levels of DHA [29-31] and choline [32,33] in the brain are affected by their plasma concentrations, supplemental dietary B-vitamins could ultimately sustain brain DHA and choline levels. This may be relevant in conditions such as Alzheimer’s disease, that are associated with lower plasma and/or brain levels of DHA [34,35], choline [36], and concurrent B-vitamin deficiencies [37,38].

## Conclusion

The present study shows that concurrently varying the dietary intake of folic acid, vitamin B-12, and vitamin B-6 can influence plasma concentration of DHA. Poor dietary intake of the three B-vitamins causes plasma DHA to decrease and this decrease can be abolished through dietary supplementation with these vitamins. Furthermore, plasma DHA concentrations can be enhanced by supplemental intake of these B-vitamins exceeding normal dietary recommendations.

## Abbreviations

CDP: Cytidine Di Phosphate; DHA: Docosahexaenoic Acid; PC: Phosphatidyl Choline; PE: Phosphatidyl Ethanolamine; PEMT: Phosphatidyl Ethanolamine-N-Methyl Transferase; PUFA: Poly Unsaturated Fatty Acid; SAH: S-Adenosyl Homocysteine; SAM: S-Adenosyl Methionine; VLDL: Very Low Density Lipoprotein.

## Competing interests

The research as described in this paper was funded by Nutricia Advanced Medical Nutrition, Danone Research, Centre for Specialised Nutrition.

Author disclosures. NvW, RJJH, JWCS, PJGHK and LMB are all employees of Danone Research, Centre for Specialised Nutrition. RJW is a scientific consultant of Danone Research, Centre for Specialised Nutrition. CJW has no competing interests.

## Authors’ contributions

The contribution of each author to the present paper was as follows: NvW, RJJH, JWCS, PJGHK, RJW and LMB were responsible for the research design; NvW and CJW conducted research; NvW performed data analysis and statistical analysis; and NvW and LMB prepared the manuscript. All co-authors read and approved the final manuscript.

## References

[B1] Li D Mann NJ Sinclair AJ A significant inverse relationship between concentrations of plasma homocysteine and phospholipid docosahexaenoic acid in healthy male subjects Lipids 2006 41 85 89 10.1007/s11745-006-5074-x 16555476

[B2] Rasmussen LE Svensson M Jorgensen KA Schmidt EB Christensen JH The content of docosahexaenoic acid in serum phospholipid is inversely correlated with plasma homocysteine levels in patients with end-stage renal disease Nutr Res 2010 30 535 540 10.1016/j.nutres.2010.07.004 20851307

[B3] Selley ML A metabolic link between S-adenosylhomocysteine and polyunsaturated fatty acid metabolism in Alzheimer’s disease Neurobiol Aging 2007 28 1834 1839 10.1016/j.neurobiolaging.2006.08.003 16996649

[B4] Durand P Prost M Blache D Pro-thrombotic effects of a folic acid deficient diet in rat platelets and macrophages related to elevated homocysteine and decreased n-3 polyunsaturated fatty acids Atherosclerosis 1996 121 231 243 10.1016/0021-9150(95)06724-8 9125297

[B5] Peifer JJ Lewis RD Effects of vitamin B-12 deprivation on phospholipid fatty acid patterns in liver and brain of rats fed high and low levels of linoleate in low methionine diets J Nutr 1979 109 2160 2172 512704 10.1093/jn/109.12.2160

[B6] Delorme CB Lupien PJ The effect of vitamin B-6 deficiency on the fatty acid composition of the major phospholipids in the rat J Nutr 1976 106 169 180 1249643 10.1093/jn/106.2.169

[B7] Kennedy EP Weiss SB The function of cytidine coenzymes in the biosynthesis of phospholipides J Biol Chem 1956 222 193 214 13366993

[B8] Vance DE Walkey CJ Cui Z Phosphatidylethanolamine N-methyltransferase from liver Biochim Biophys Acta 1997 1348 142 150 10.1016/S0005-2760(97)00108-2 9370326

[B9] Watkins SM Zhu X Zeisel SH Phosphatidylethanolamine-N-methyltransferase activity and dietary choline regulate liver-plasma lipid flux and essential fatty acid metabolism in mice J Nutr 2003 133 3386 3391 14608048 10.1093/jn/133.11.3386

[B10] Pynn CJ Henderson NG Clark H Koster G Bernhard W Postle AD Specificity and rate of human and mouse liver and plasma phosphatidylcholine synthesis analyzed in vivo J Lipid Res 2011 52 399 407 10.1194/jlr.D011916 21068006 PMC3023562

[B11] Yao ZM Vance DE The active synthesis of phosphatidylcholine is required for very low density lipoprotein secretion from rat hepatocytes J Biol Chem 1988 263 2998 3004 3343237

[B12] Noga AA Zhao Y Vance DE An unexpected requirement for phosphatidylethanolamine N-methyltransferase in the secretion of very low density lipoproteins J Biol Chem 2002 277 42358 42365 10.1074/jbc.M204542200 12193594

[B13] Noga AA Vance DE A gender-specific role for phosphatidylethanolamine N-methyltransferase-derived phosphatidylcholine in the regulation of plasma high density and very low density lipoproteins in mice J Biol Chem 2003 278 21851 21859 10.1074/jbc.M301982200 12668679

[B14] DeLong CJ Shen YJ Thomas MJ Cui Z Molecular distinction of phosphatidylcholine synthesis between the CDP-choline pathway and phosphatidylethanolamine methylation pathway J Biol Chem 1999 274 29683 29688 10.1074/jbc.274.42.29683 10514439

[B15] Tacconi M Wurtman RJ Phosphatidylcholine produced in rat synaptosomes by N-methylation is enriched in polyunsaturated fatty acids Proc Natl Acad Sci U S A 1985 82 4828 4831 10.1073/pnas.82.14.4828 3860825 PMC390998

[B16] da Costa KA Rai KS Craciunescu CN Parikh K Mehedint MG Sanders LM McLean-Pottinger A Zeisel SH Dietary docosahexaenoic acid supplementation modulates hippocampal development in the Pemt−/− mouse J Biol Chem 2010 285 1008 1015 10.1074/jbc.M109.017137 19889625 PMC2801227

[B17] Reeves PG Nielsen FH Fahey GC AIN-93 purified diets for laboratory rodents: final report of the American Institute of Nutrition ad hoc writing committee on the reformulation of the AIN-76A rodent diet J Nutr 1993 123 1939 1951 8229312 10.1093/jn/123.11.1939

[B18] Cullen RW Oace SM Dietary pectin shortens the biologic half-life of vitamin B-12 in rats by increasing fecal and urinary losses J Nutr 1989 119 1121 1127 2550599 10.1093/jn/119.8.1121

[B19] Hove EL King S Effects of pectin and cellulose on growth, feed efficiency, and protein utilization, and their contribution to energy requirement and cecal VFA in rats J Nutr 1979 109 1274 1278 448468 10.1093/jn/109.7.1274

[B20] National Research Council Nutrient requirements of laboratory animals, Fourth Revised Edition edn 1995 National Academic Press, Washington 25121259

[B21] Krijt J Vackova M Kozich V Measurement of homocysteine and other aminothiols in plasma: advantages of using tris(2-carboxyethyl)phosphine as reductant compared with tri-n-butylphosphine Clin Chem 2001 47 1821 1828 11568092

[B22] Yi P Melnyk S Pogribna M Pogribny IP Hine RJ James SJ Increase in plasma homocysteine associated with parallel increases in plasma S-adenosylhomocysteine and lymphocyte DNA hypomethylation J Biol Chem 2000 275 29318 29323 10.1074/jbc.M002725200 10884384

[B23] da Costa KA Sanders LM Fischer LM Zeisel SH Docosahexaenoic acid in plasma phosphatidylcholine may be a potential marker for in vivo phosphatidylethanolamine N-methyltransferase activity in humans Am J Clin Nutr 2011 93 968 974 10.3945/ajcn.110.011064 21411618 PMC3076652

[B24] Pita ML Delgado MJ Folate administration increases n-3 polyunsaturated fatty acids in rat plasma and tissue lipids Thromb Haemost 2000 84 420 423 11019965

[B25] Krauss-Etschmann S Shadid R Campoy C Hoster E Demmelmair H Jimenez M Gil A Rivero M Veszpremi B Decsi T Koletzko BV Effects of fish-oil and folate supplementation of pregnant women on maternal and fetal plasma concentrations of docosahexaenoic acid and eicosapentaenoic acid: a European randomized multicenter trial Am J Clin Nutr 2007 85 1392 1400 17490978 10.1093/ajcn/85.5.1392

[B26] Crowe FL Skeaff CM McMahon JA Williams SM Green TJ Lowering plasma homocysteine concentrations of older men and women with folate, vitamin B-12, and vitamin B-6 does not affect the proportion of (n-3) long chain polyunsaturated fatty acids in plasma phosphatidylcholine J Nutr 2008 138 551 555 18287365 10.1093/jn/138.3.551

[B27] Umhau JC Dauphinais KM Patel SH Nahrwold DA Hibbeln JR Rawlings RR George DT The relationship between folate and docosahexaenoic acid in men Eur J Clin Nutr 2006 60 352 357 10.1038/sj.ejcn.1602321 16278690

[B28] van Wijk N Watkins CJ Bohlke M Maher TJ Hageman RJ Kamphuis PJ Broersen LM Wurtman RJ Plasma choline concentration varies with different dietary levels of vitamins B6, B12 and folic acid in rats maintained on choline-adequate diets Br J Nutr 2012 107 1408 1412 10.1017/S0007114511004570 21917195

[B29] Rapoport SI Chang MC Spector AA Delivery and turnover of plasma-derived essential PUFAs in mammalian brain J Lipid Res 2001 42 678 685 11352974

[B30] Brossard N Croset M Lecerf J Pachiaudi C Normand S Chirouze V Macovschi O Riou JP Tayot JL Lagarde M Metabolic fate of an oral tracer dose of [13 C]docosahexaenoic acid triglycerides in the rat Am J Physiol 1996 270 R846 R854 8967415 10.1152/ajpregu.1996.270.4.R846

[B31] Connor WE Neuringer M Lin DS Dietary effects on brain fatty acid composition: the reversibility of n-3 fatty acid deficiency and turnover of docosahexaenoic acid in the brain, erythrocytes, and plasma of rhesus monkeys J Lipid Res 1990 31 237 247 2139096

[B32] Klein J Koppen A Loffelholz K Small rises in plasma choline reverse the negative arteriovenous difference of brain choline J Neurochem 1990 55 1231 1236 10.1111/j.1471-4159.1990.tb03129.x 2398357

[B33] Cohen EL Wurtman RJ Brain acetylcholine: control by dietary choline Science 1976 191 561 562 10.1126/science.1251187 1251187

[B34] Conquer JA Tierney MC Zecevic J Bettger WJ Fisher RH Fatty acid analysis of blood plasma of patients with Alzheimer’s disease, other types of dementia, and cognitive impairment Lipids 2000 35 1305 1312 10.1007/s11745-000-0646-3 11201991

[B35] Soderberg M Edlund C Kristensson K Dallner G Fatty acid composition of brain phospholipids in aging and in Alzheimer’s disease Lipids 1991 26 421 425 10.1007/BF02536067 1881238

[B36] Nitsch RM Blusztajn JK Pittas AG Slack BE Growdon JH Wurtman RJ Evidence for a membrane defect in Alzheimer disease brain Proc Natl Acad Sci U S A 1992 89 1671 1675 10.1073/pnas.89.5.1671 1311847 PMC48514

[B37] Glaso M Nordbo G Diep L Bohmer T Reduced concentrations of several vitamins in normal weight patients with late-onset dementia of the Alzheimer type without vascular disease J Nutr Health Aging 2004 8 407 413 15359361

[B38] Koseoglu E Karaman Y Relations between homocysteine, folate and vitamin B12 in vascular dementia and in Alzheimer disease Clin Biochem 2007 40 859 863 10.1016/j.clinbiochem.2007.04.007 17532313

